# Mitophagy in fibrotic diseases: molecular mechanisms and therapeutic applications

**DOI:** 10.3389/fphys.2024.1430230

**Published:** 2024-08-09

**Authors:** Xinyan Cui, Zekun Zhou, Hua Tu, Jianjun Wu, Jian Zhou, Qiao Yi, Ousheng Liu, Xiaohan Dai

**Affiliations:** ^1^ Hunan Key Laboratory of Oral Health Research, Hunan Clinical Research Center of Oral Major Diseases, Oral Health and Academician Workstation for Oral-maxilofacial, Regenerative Medicine and Xiangya Stomatological Hospital, Xiangya School of Stomatology, Central South University, Changsha, Hunan, China; ^2^ Salivary Gland Disease Center and Beijing Key Laboratory of Tooth Regeneration and Function Reconstruction, Beijing Laboratory of Oral Health and Beijing Stomatological Hospital, Capital Medical University, Beijing, China; ^3^ Department of VIP Dental Service, School of Stomatology, Capital Medical University, Beijing, China; ^4^ Laboratory for Oral and General Health Integration and Translation, Beijing Tiantan Hospital, Capital Medical University, Beijing, China

**Keywords:** mitophagy, fibrosis, oxidative stress, inflammation, energy metabolism, treatment

## Abstract

Mitophagy is a highly precise process of selective autophagy, primarily aimed at eliminating excess or damaged mitochondria to maintain the stability of both mitochondrial and cellular homeostasis. In recent years, with in-depth research into the association between mitophagy and fibrotic diseases, it has been discovered that this process may interact with crucial cellular biological processes such as oxidative stress, inflammatory responses, cellular dynamics regulation, and energy metabolism, thereby influencing the occurrence and progression of fibrotic diseases. Consequently, modulating mitophagy holds promise as a therapeutic approach for fibrosis. Currently, various methods have been identified to regulate mitophagy to prevent fibrosis, categorized into three types: natural drug therapy, biological therapy, and physical therapy. This review comprehensively summarizes the current understanding of the mechanisms of mitophagy, delves into its biological roles in fibrotic diseases, and introduces mitophagy modulators effective in fibrosis, aiming to provide new targets and theoretical basis for the investigation of fibrosis-related mechanisms and disease prevention.

## 1 Introduction

**FIGURE 1:**
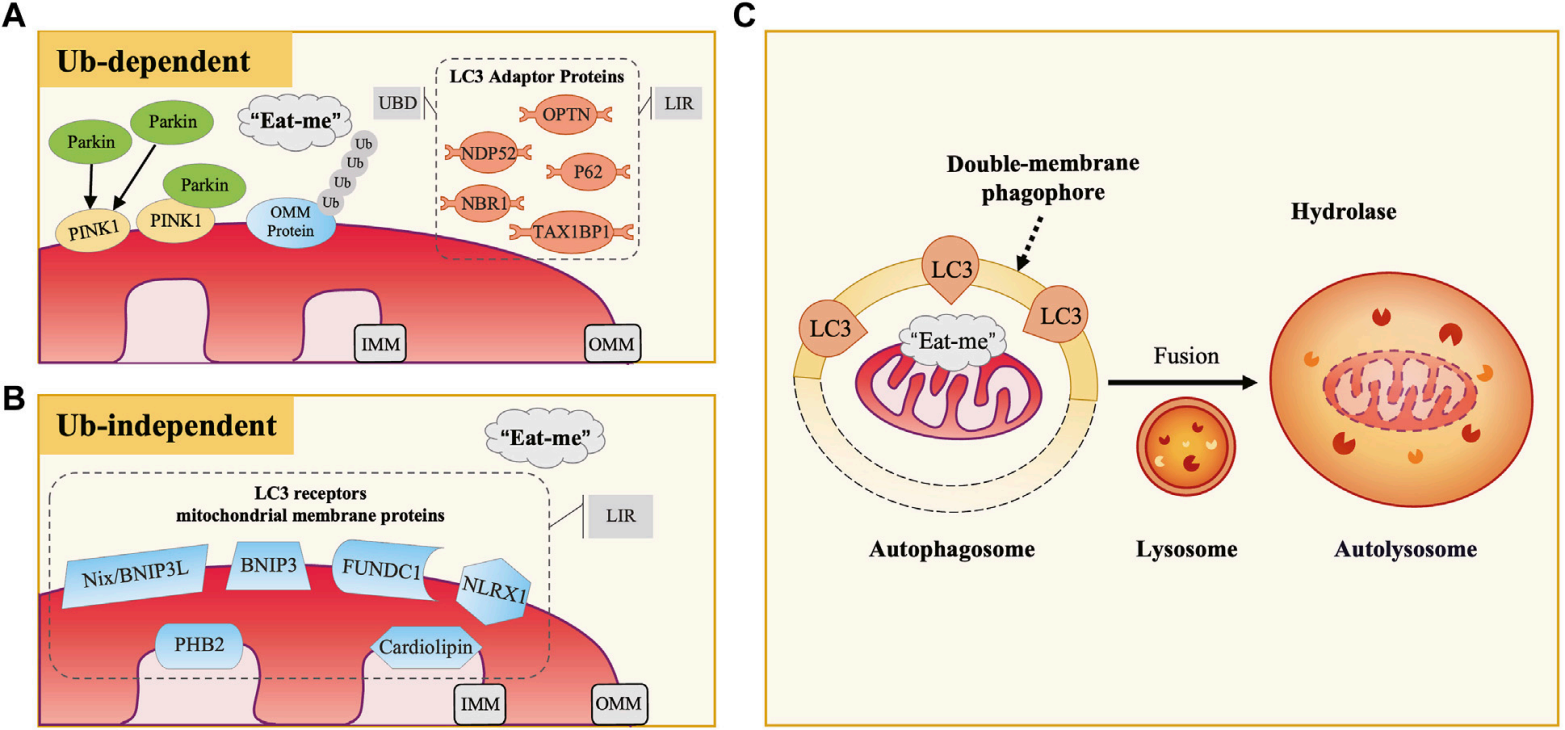
The Occurrence and Mechanisms of Mitophagy. **(A)** Ubiquitin-Dependent Mitophagy: PINK1/Parkin Pathway. In defective mitochondria, PINK1 accumulates on the outer mitochondrial membrane (OMM), recruiting and activating Parkin, which ubiquitinates OMM proteins. LC3 adaptor proteins (e.g., P62, OPTN, NDP52) bind to these ubiquitinated proteins via UBD motifs and interact with LC3 through LIR motifs, facilitating the engulfment of damaged mitochondria. **(B)** Ubiquitin-Independent Mitophagy. This type of mitophagy involves specific mitochondrial membrane protein receptors like Nix/BNIP3L, BNIP3, and FUNDC1. These receptors directly bind to LC3 via their LIR motifs, acting as LC3 receptors. **(C)** Common Mitophagy Process. Both mechanisms involve the release of “eat me” signals from defective mitochondria. LC3 guides the mitochondria to be engulfed by double-membrane autophagosomes, forming autophagosomes. These then fuse with lysosomes to form autolysosomes, where mitochondria are degraded by lysosomal hydrolases.

Mitochondria are crucial cellular organelles involved in energy metabolism, signal transduction, and the maintenance of cellular homeostasis (Zhao et al., 2020). Under physiological conditions, mitochondria exhibit a sensitive response to cellular stress and metabolic changes. However, when confronted with noxious stimuli, mitochondria can release large amounts of mitochondrial reactive oxygen species (mtROS), a byproduct of oxidative phosphorylation. The release of these ROS triggers oxidative stress (OS), a condition characterized by an imbalance between the production of ROS and the body’s ability to detoxify these reactive intermediates or repair the resulting damage, leading to mitochondrial dysfunction. Additionally, under the attack of ROS, mitochondrial DNA (mtDNA) is damaged due to the lack of histone protection and released into the cytoplasm, triggering inflammatory and immune responses and further adversely affecting cells (Topf et al., 2016). To maintain cell health, eukaryotic organisms have evolved a self-degradative process specific to the mitochondria, known as mitophagy. Its occurrence includes four key steps: 1) in response to stimuli such as ROS, nutrient starvation, or cellular senescence, mitochondria undergo depolarization and reduction of mitochondria membrane potential (∆Ψm), indicating the loss of normal physiological functions; 2) damaged mitochondria accumulate specific membrane proteins or receptors, potentially forming ubiquitin chains, thereby releasing “eat me” signals that prompt the cell to initiate mitophagy, with autophagosome membrane proteins recognizing and binding to these signals, subsequently encapsulating the mitochondria within double-membrane phagophores to form autophagosomes; 3) autophagosomes fuse with lysosomes to form autolysosomes, where mitochondria are delivered for degradation; 4) lysosomal hydrolases enter autolysosomes, effectively degrading and recycling components within mitochondria (Picca et al., 2021) (Figure 1C). As a pivotal mechanism for mitochondrial quality control, mitophagy not only selectively eliminates dysfunctional mitochondria characterized by low ΔΨm and high ROS levels, thereby reducing the release of harmful substances and preventing cell death, but also recycles amino acids from mitochondria to synthesize new proteins and maintain normal metabolic levels. This intricate and effective intracellular regulatory mechanism contributes to maintaining the mitochondrial network healthy, restoring cellular homeostasis, and providing essential safeguards for cells to adapt to external environmental changes.

Fibrosis remains a significant contributor of increased mortality on a global scale. It is the result of a dysregulated repair response to tissue damage and can occur in vital organs such as the heart, lung, liver, and kidney, as well as in tissues like the skin, mucous membrane, pancreatic islet, and even bone marrow (Henderson et al., 2020; Xu et al., 2020). Common diseases associated with fibrosis include chronic heart failure (CHF), idiopathic pulmonary fibrosis (IPF), non-alcoholic fatty liver disease (NAFLD), cirrhosis, and chronic kidney disease (CKD), characterized by high prevalence, elevated mortality rates, and substantial healthcare costs (Li et al., 2020). According to research data from 2020, the comprehensive annual incidence of major fibrotic diseases globally is approximately 4,968 cases per 100,000 person-years, imposing an enormous burden on both health and the economy, and so far, there are no preventive or reversal therapies for fibrosis (Zhao et al., 2022). Therefore, fibrotic diseases are considered a formidable health challenge, necessitating urgent exploration of new therapeutic targets to improve the treatment landscape.

Studies have revealed that many fibrotic diseases exhibit mitochondrial fragment accumulation or dysfunction, suggesting that abnormalities in mitochondrial clearance mechanisms are closely associated with fibrosis (Higgins and Coughlan, 2014; Roque et al., 2020; Cai et al., 2022). Besides, modulation of mitophagy levels has been proved to affect the degree of fibrosis (Bueno et al., 2015; Song et al., 2015; Ronggjun et al., 2024). Therefore, gaining insight into the link between mitophagy and the dynamic cellular and molecular events of fibrosis should be a current research priority. In this review, we provided a detailed description of the mechanisms of mitophagy in fibrotic diseases, particularly its interactions with cellular biological processes such as OS, inflammation, cellular dynamics regulation, and energy metabolism. This serves as a theoretical basis for further probing into the mechanisms underlying fibrosis. Furthermore, we proposed that intervention targeting mitophagy may be an effective approach for treating fibrosis. We focused on introducing currently discovered methods for modulating mitophagy, categorizing them into three classes: natural medicine therapy, biotherapy, and physiotherapy. Delving into the mechanisms and potential applications of these methods is promising to bring breakthroughs in fibrotic disease treatment.

## 2 Mechanisms of mitophagy

Over the years, studies of mitophagy mechanisms in mammalian cells have been primarily classified into two categories: ubiquitin-dependent and ubiquitin-independent pathways. Microtubule-associated protein-1 light chain-3 (MAP1LC3), abbreviated as LC3, is a subfamily of the autophagy-related gene 8 (Atg8) family in eukaryotes, which includes LC3A, LC3B, LC3B2, and LC3C (Shpilka et al., 2011). Among them, LC3B (hereafter referred to as LC3) is the most common and extensively studied marker on autophagosome membranes, playing a crucial role in autophagosome formation, membrane extension, and cargo recognition. It undergoes conversion from its cytosolic LC3-I form to the membrane-bound LC3-II form, marking a critical stage in the autophagic process (Weidberg et al., 2011). According to the binding mode of mitochondria to the autophagosome membrane, it can be further classified into two categories, LC3 adaptor proteins (indirect way) and LC3 receptors (direct way). In this section, we will provide a detailed overview of these critical autophagy proteins and the mechanisms of mitophagy.

### 2.1 LC3 adaptor protein and ubiquitin-dependent pathway

Currently, research has identified five LC3 adaptor proteins, including sequestosome-1 (SQSTM1/P62), optineurin (OPTN), nuclear dot protein 52 (NDP52), TAX1 binding protein 1 (TAX1BP1), and neighbor of breast cancer susceptibility gene one protein (NBR1) (Lazarou et al., 2015). During the process of mitophagy, LC3 adaptor proteins exert a bridge-like connecting role, binding to ubiquitinated cargo through the ubiquitin-binding domain (UBD) motif on one side, recognizing and binding to LC3 on the autophagosome membrane through the LC3-interacting region (LIR) motif on the other side (Stolz et al., 2014) (Figure 1A).

#### 2.1.1 PINK1/parkin-mediated mitophagy

PTEN-induced putative kinase 1 (PINK1) is an outer mitochondrial membrane (OMM) kinase, whereas Parkin is an E3 ubiquitin ligase present in the cytosol, both of which are gene products mutated in Parkinson’s disease (Valente et al., 2004; Pickrell and Youle, 2015). The PINK1/Parkin pathway plays an essential role in the regulation of mammalian cellular mitophagy and is currently the best-characterized and most extensively studied ubiquitin-dependent pathway (Eiyama and Okamoto, 2015). Physiologically, PINK1 is translocated from OMM to inner mitochondrial membrane (IMM), where its mitochondrial targeting sequence (MTS) is excised by matrix processing peptidase (MPP), subsequently cleaved by PINK1/PGAM5-associated rhomboid-like protease (PARL) and ultimately degraded in the cytoplasm by the proteasome (Jin et al., 2010; Greene et al., 2012; Yamano and Youle, 2013). Therefore, the content of PINK1 in normal mitochondria and the cytoplasm is minimal. When external stimuli cause damage to mitochondria, the decrease of ∆Ψm inhibits PINK1 hydrolysis, leading to persistent and massive presence of PINK1 on the OMM. Through phosphorylation of nearby ubiquitin molecules, PINK1 recruits and activates Parkin (Narendra et al., 2008; Matsuda et al., 2010; Narendra et al., 2010). Activated Parkin promotes ubiquitination of OMM proteins, constructing ubiquitin chains that act as “eat me” signals for damaged mitochondria (Eiyama and Okamoto, 2015; Nguyen et al., 2016; Pickles et al., 2018). Subsequently, LC3 adaptor proteins (especially OPTN and NDP52) recognize ubiquitination signals and target damaged mitochondria to autophagosomes, thereby inducing the occurrence of mitophagy.

#### 2.1.2 PINK1/non-parkin-mediated mitophagy

Research has also revealed the existence of PINK1/non-Parkin-mediated mitophagy, in which PINK1 directly recruits LC3 adaptor proteins OPTN and NDP52 to mitochondria, thereby activating mitophagy independent of Parkin (Lazarou et al., 2015). Additionally, PINK1 recruits other E3 ubiquitin ligases apart from Parkin, facilitating mitochondrial protein ubiquitination and inducing mitophagy.

### 2.2 LC3 receptor and ubiquitin-independent pathway

Some mitochondrial membrane protein receptors have putative LIR motifs that directly bind to LC3. Therefore, they can also act as “eat me” signals when mitochondria are damaged, inducing mitophagy without depending on ubiquitination. In mammalian cells, currently recognized LC3 receptors on the mitochondrial membrane include nip-like protein X (NIX)/B-cell lymphoma two interacting protein three like (BNIP3L) and BNIP3 family, FUN14 domain-containing 1 (FUNDC1), NOD-like receptor X1 (NLRX1), prohibitin 2 (PHB2), and cardiolipin, among others (Liu et al., 2014) (Figure 1B). Under different experimental conditions, these receptors have been demonstrated to directly participate in mitophagy.

The OMM proteins Nix/BNIP3L and BNIP3 are homologous and can induce mitophagy by directly binding to LC3 (Matsushima et al., 1998; Novak et al., 2010). Early studies found that NIX/BNIP3L played a role in the mitophagy process essential for reticulocyte maturation and was anchored on the mitochondrial surface in the late stage of erythrocyte maturation (Aerbajinai et al., 2003; Sandoval et al., 2008). Additionally, hypoxia-inducible factor 1-alpha (HIF-1α)-mediated hypoxia induced upregulation of BNIP3 and NIX/BNIP3L, which activated cell death pathways in solid tumors (Sowter et al., 2001).

FUNDC1 is also an OMM protein, and its conserved Y18 and L21 in the sequence are essential for the interaction between FUNDC1 and LC3 (Liu et al., 2012). Research has shown that under hypoxic stress, the inactivation of Src kinase and casein kinase II, along with the activation of phosphoglycerate mutase family member 5 (PGAM5), significantly enhanced the activity of FUNDC1 and its interaction with LC3, thereby activating mitophagy (Chen et al., 2014). Upregulation of FUNDC1-mediated mitophagy further activated the ROS/HIF-1α pathway, participating in the development of hypoxia-associated diseases (Liu et al., 2022).

NLRX1 participated in the mitophagy response to *Listeria* monocytogenes infection by targeting LC3 to damaged mitochondria independently of PINK1 and Parkin (Zhang et al., 2019). Furthermore, when mitochondrial depolarization caused mitochondrial protein import stress, NLRX1 recruited ribosome binding protein 1 (RRBP1) of the endoplasmic reticulum (ER) membrane to form the NLRX1/RRBP1 complex, which induced LC3 recruitment and lipidation, and consequently affected the formation of mitochondrial autophagosomes (Killackey et al., 2023; 2022). NLR family proteins play a crucial role in innate immunity. Research has revealed that NLRX1 exerted negative regulatory effects in mitochondrial antiviral signaling protein (MAVS)-mediated anti-viral signal transduction, thus participating in host innate immune processes (Song et al., 2019).

Unlike other LC3 receptors, PHB2 and cardiolipin receptors are both IMM proteins. Among them, PHB2 is a crucial receptor for Parkin-mediated mitophagy in mammalian cells. It has been identified that after Parkin-mediated proteasomal degradation of the OMM, PHB2 on the IMM was exposed as a direct target, with LC3 binding to the LIR motif of PHB2, thereby inducing mitophagy (Wei Y. et al., 2017). Another study specifically elucidated its mechanism of action, suggesting that the PHB2 receptor promoted mitophagy through the PHB2-PARL-PGAM5-PINK1 axis (Yan et al., 2020). However, it remains unclear how the PHB2 receptors on the IMM are exposed in Parkin-independent mitophagy. Furthermore, the use of 6-hydroxydopamine and rotenone induced the translocation of cardiolipin to the OMM in neuronal cells, promoting the binding of LC3 to cardiolipin and the occurrence of mitophagy (Chu et al., 2013).

## 3 Interactions between mitophagy and fibrotic processes

Fibrosis is a pathological remodeling caused by an excessive tissue repair response (Henderson et al., 2020). The mechanism involves initial parenchymal cell death and activation of the inflammatory response at the damaged site, leading to the recruitment of numerous immune cells that produce various cytokines and chemokines. This promotes the transition of fibroblasts (FB), epithelial cells, and others into myofibroblasts (MFB). Once activated, MFB synthesize and secrete extracellular matrix (ECM) components, which deposit in the damaged and interstitial tissues, thereby initiating the repair response and promoting wound healing (Wynn, 2008; Rockey et al., 2015). If the tissue damage is brief and mild, the transient increase in ECM deposition can restore functional structure efficiently. However, if the damage is prolonged, recurrent, or severe, the damaged cells undergo chronic inflammation, oxidative stress, and senescence, leading to a sustained and excessive repair response. This ultimately results in irreversible fibrosis, causing organ dysfunction and even failure (Henderson et al., 2020).

Research has underscored a crucial interplay between defective mitophagy and the progression of fibrosis. Specifically, PINK1 gene ablation promoted pulmonary fibrogenesis by impairing mitochondrial homeostasis (Bueno et al., 2015; Meredith et al., 2015); or aggravated kidney injury and fibrosis by increasing cellular senescence (Min et al., 2023). In comparison, Parkin gene ablation exacerbated premature aging, inflammation, and fibrotic lesions in diabetic nephropathy (DN) mice (Kehong et al., 2020); attenuated regeneration of damaged skeletal muscle and increase fibrotic area (Marcos et al., 2020); increased susceptibility to hepatic steatosis and fibrosis (Edmunds et al., 2020; Undamatla et al., 2023); and, unlike the former, inhibited over-activated mitophagy, exerting an anti-cardiac fibrosis effect (Song et al., 2015). Mitophagy plays a pivotal role in modulating fibrotic diseases by influencing crucial cellular processes such as OS, inflammation, cellular dynamics, and energy metabolism, which further affects the activation of MFB and fibrosis-related factors, as well as the synthesis and secretion of ECM. However, the effects and regulatory mechanisms of mitophagy vary in different disease sites, pathological stages, and triggering conditions. Therefore, in this section, we will focus on exploring the molecular regulatory mechanisms of mitophagy in fibrotic diseases.

### 3.1 Interaction of mitophagy and OS in fibrotic diseases

**FIGURE 2:**
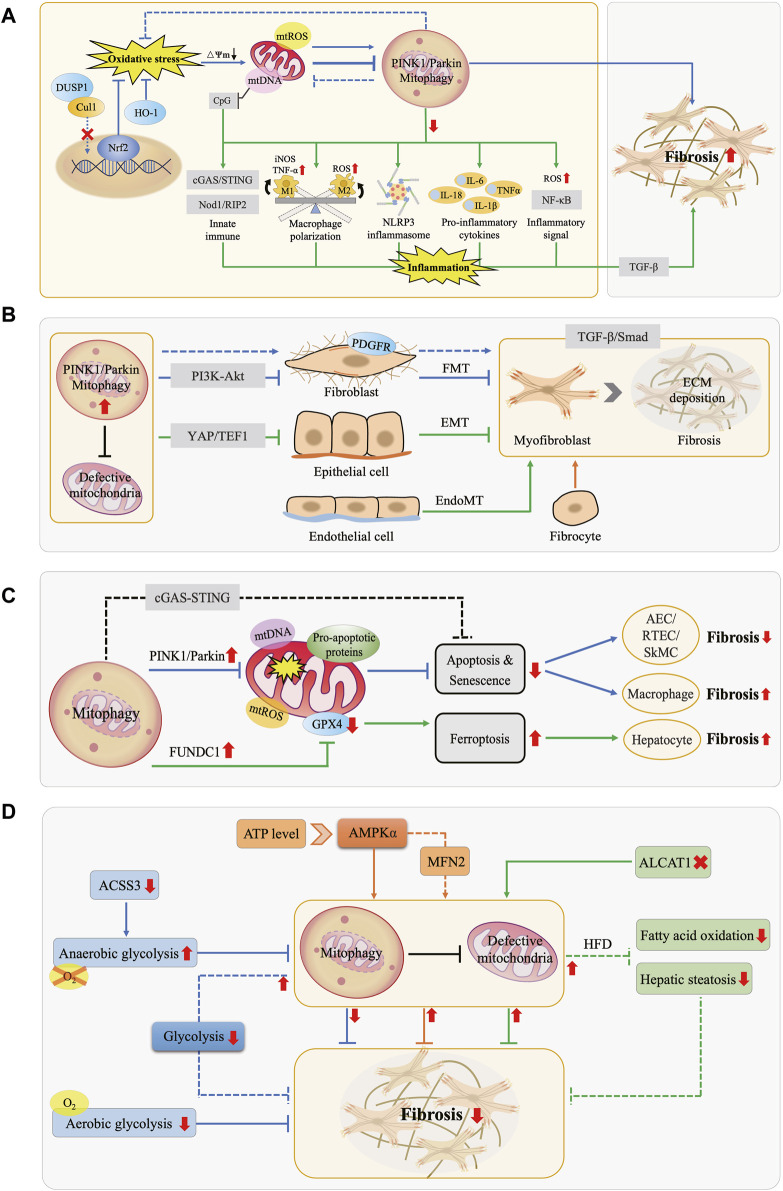
Interactions Between Mitophagy and Fibrotic Processes. **(A)** Mitophagy, OS, and Inflammation in Fibrosis. Under physiological conditions, mitophagy mitigates OS and maintains cellular homeostasis. OS can enhance or inhibit mitophagy, promoting fibrosis. Downregulation of mitophagy activates the NLRP3 inflammasome, releases pro-inflammatory factors, elevates NF-κB activity, modulates macrophage M1/M2 polarization, and leads to mtDNA cytoplasmic leakage, triggering the innate immune system. These effects collectively exacerbate inflammation and fibrosis. **(B)** Mitophagy and MFB Activation in Fibrosis. MFB primarily originate from FMT, EMT, EndoMT, and bone marrow-derived fibrocytes. Mitophagy hinders MFB activation and ECM deposition by inhibiting the PDGFR-PI3K-Akt, YAP/TEF1, and TGF-β/Smad pathways, counteracting fibrosis. **(C)** Mitophagy and Cell Death in Fibrosis. Mitophagy exerts an anti-fibrotic effect by inhibiting apoptosis in tissue-intrinsic cells such as AEC, RTEC, and SMC, while it manifests as promoting fibrosis in macrophages. Moreover, FUNDC1-mediated mitophagy leads to ferroptosis, thus promoting fibrosis. **(D)** Mitophagy and Energy Metabolism in Fibrosis. AMPK molecules perceive energy changes and enhance mitophagy, while increasing anaerobic glycolysis or decreasing aerobic glycolysis inhibits mitophagy, both of which act as anti-fibrotic effects. In turn, activation of mitophagy attenuates glycolysis in FB, inhibits fatty acid oxidation and steatosis, similarly exerting anti-fibrotic effects.

OS is typically induced by external factors such as dietary excess or deficiency, environmental toxins, and internal factors including chronic inflammation, metabolic disorders, and cellular aging, leading to massive ROS generation, which may originate from uncontrolled sources such as overstimulation of NAD(P)H oxidase or the IMM respiratory chain (Dröge, 2002). During electron transfer in mitochondria, leaked electrons continuously reduce oxygen to various forms of ROS, including the hydroxyl radical (·OH), superoxide anion (O_2_^−^·), and hydrogen peroxide (H_2_O_2_), with ·OH exhibiting the highest oxidative potential (Chen et al., 2012; Chen and Zweier, 2014). Excessive ROS affect mitochondrial structure and function by damaging mitochondrial membrane lipids and proteins, interfering with the electron transport chain, lowering ∆Ψm, and disrupting calcium homeostasis, thereby exacerbating oxidative damage, and triggering cellular stress responses. Simultaneously, mitophagy, by inhibiting ROS generation, regulating antioxidant enzyme expression, and activating antioxidant pathways, contributes to alleviating OS, improving intracellular redox balance, and maintaining cellular homeostasis and function (Figure 2A).

By summarizing relevant studies, we found that OS can lead to the undesirable consequences of fibrosis by modulating the level of mitophagy. However, it remains controversial whether mitophagy is enhanced or inhibited by OS, and whether its role is beneficial (“friend”) or detrimental (“foe”) in fibrosis. On the one hand, some studies suggest that OS enhances mitophagy, exacerbating fibrosis and thus portraying mitophagy as a “foe” in this process. For example, mitochondrial OS in alveolar macrophages induced mitophagy, which directly correlated with pulmonary fibrosis (Larson-Casey et al., 2016). Particulate matter (PM)-induced excess ROS activated PINK1/Parkin-mediated mitophagy, promoting hepatic fibrosis (Qiu et al., 2019), thereby demonstrating the negative role of mitophagy. Similarly, obesity-related OS exacerbated mitochondrial oxidative damage in the hypertensive heart, enhancing mitophagy and consequently promoting myocardial fibrosis and functional impairment (Zhang et al., 2015). Moreover, inhibition of Akt1-mediated ROS production in Kupffer cells blocked PINK1/Parkin-mediated mitophagy activation, contributing to the regression of carbon tetrachloride (CCL4)-induced hepatic fibrosis (Wu et al., 2020). On the other hand, more studies support the perspective that OS inhibits mitophagy to promote fibrosis, considering mitophagy a “friend”. For instance, H_2_O_2_-induced OS suppressed mitophagy in mouse myocardial cells (Wang F. et al., 2022); conversely, enhancing mitophagy in vascular endothelial cells reduced mtROS generation, promoted vascular formation, and alleviated myocardial fibrosis (Wei T. et al., 2017), confirming the protective role of mitophagy. Research has summarized that failure of the quality control network (ubiquitin-proteasome system, autophagy, mitophagy, etc.) of alveolar epithelial cells (AEC), a metabolically active population of lung cells, finally triggers fibrotic remodeling of lung tissues (Jeremy and Michael, 2020). Lipopolysaccharide (LPS)-induced OS in AEC inhibited mitophagy, promoting pulmonary fibrosis progression by inducing epithelial-mesenchymal transition (EMT) (Tian et al., 2022). Additionally, ER stress in AECII cells inhibited PINK1-mediated mitophagy, leading to extracellular release of mtDNA, which activated toll-like receptor 9 (TLR9) and triggered transforming growth factor-β (TGF-β) secretion, further increasing susceptibility to pulmonary fibrosis (Bueno et al., 2019).

Nuclear factor E2-related factor 2 (Nrf2) acts as an anti-OS sensor that activates antioxidant responses within cells and is a crucial regulator of mitophagy, thereby inhibiting fibrosis by maintaining mitochondrial mass and function. Studies have shown that inactivation of Nrf2, either through direct knockdown in CKD or indirect knockdown of endothelial nitric oxide synthase (eNOS) in hepatocytes, exacerbated OS and impaired PINK1/Parkin-mediated mitophagy, contributing to the progression of renal and hepatic fibrosis (Sheldon et al., 2019; Ma et al., 2021). Recent research has also identified that certain OS-related enzymes play a role in the fibrosis process by affecting mitophagy. For instance, in the alcohol-induced OS state, low expression of dual-specificity phosphatase-1 (DUSP1) caused translocation of the E3 ubiquitin ligase component cullin-1 (CUL1) to the nucleus, leading to defective Parkin-mediated mitophagy and triggering hepatic fibrosis (Li R. et al., 2023). Additionally, the antioxidant heme oxygenase-1 (HO-1) is upregulated under various stress stimuli, exerting a role in mitigating OS and reducing tissue damage. However, in mice with specific knockout of the HO-1 gene, sustained cardiac OS induced by the hyperoxia environment inhibited the binding of nuclear respiratory factor 1 (NRF-1) to Pink1/Parkin gene locus, resulting in defective mitophagy, increased cell death, extensive collagen deposition, ultimately triggering myocardial injury and fibrosis (Suliman et al., 2017).

### 3.2 Mitophagy mediated inflammation in fibrotic diseases

Chronic inflammation is a key feature of fibrotic diseases, characterized by the activation of inflammasomes such as NLR family pyrin domain containing 3 (NLRP3), inflammatory signaling pathways like nuclear factor-kappa B (NF-κB), and the upregulation of pro-inflammatory cytokines, along with the recruitment and polarization of immune cells. Damaged mitochondria release pro-inflammatory substances like mtROS, mtDNA, and cardiolipin, promoting fibrosis progression. In the inflammatory state, mitophagy acts as a protective mechanism by removing damaged mitochondria and pro-inflammatory substances, thereby exerting anti-inflammatory and anti-fibrotic effects (Figure 2A). Conversely, defective mitophagy hastened the occurrence of tissue inflammation and fibrosis (Undamatla et al., 2023).

Mitophagy plays a crucial role in the pathogenesis of fibrosis by regulating inflammatory manifestations. Firstly, dysfunction in mitophagy exacerbates the inflammatory response and fibrosis by activating the inflammasomes and pro-inflammatory factors. For example, inhibition of mitophagy has been shown to induce NLRP3 inflammasome activation and IL-18 overexpression, leading to myocardial fibrosis (Liang et al., 2023). Similarly, inhibition of PINK1/Parkin-mediated mitophagy promoted the progression of NAFLD by activating the NLRP3 inflammasome and triggering cellular pyroptosis, a distinct form of programmed cell death characterized by inflammation, cell swelling, and rupture (Gao et al., 2022). In non-alcoholic steatohepatitis (NASH), inhibition of parkin-mediated mitophagy led to the accumulation of dysfunctional mitochondria and increased production of the pro-inflammatory cytokine TNF-α by macrophages, creating a positive feedback loop that further inhibited mitophagy and exacerbated hepatic steatosis and fibrosis (Jin et al., 2023). Additionally, impaired mitophagy in muscle fibers aggravated muscle damage and fibrosis by activating pro-inflammatory factors like IL-1β and IL-6 (Reid and Alexander, 2021). Secondly, mitophagy regulates key inflammatory signaling molecules such as NF-κB to participate in the fibrotic process. In hypertensive mice with deficient lysosomal cathepsin S in macrophages, mitophagy was inhibited, resulting in elevated ROS levels and NF-κB activity, exacerbating cardiac inflammation and fibrosis (Pan et al., 2012). Conversely, upregulation of mitophagy ameliorated renal fibrosis by inhibiting the activation of NF-κB and TGF-β1 signaling, reducing cellular damage and inflammatory infiltration (Jin et al., 2022). Lastly, mitophagy plays a vital role in macrophage polarization, a key immunological mechanism of fibrogenesis. When mitophagy was impaired in DN, macrophages tended to shift towards the pro-inflammatory M1 phenotype, accompanied by increased expression of inducible nitric oxide synthase (iNOS) and TNF-α. Activation of mitophagy significantly inhibited the M1 phenotype and promoted a transition to the anti-inflammatory M2 phenotype (Zhao et al., 2017). Another study found that downregulation of mitofusin 2 (MFN2) led to macrophage recruitment to the kidney and polarization towards the pro-fibrotic M2 phenotype by enhancing OS and inhibiting macrophage mitophagy, thereby promoting collagen deposition and renal fibrosis (Bhatia et al., 2022).

Mitophagy also modulates inflammation-mediated fibrosis by influencing the innate immune system. Sustained stress stimuli that impair mitophagy lead to the accumulation of damaged mitochondria and the release of mtDNA, which, due to its bacterial-like CpG islands, is recognized as a damage-associated molecular pattern (DAMP) by the innate immune system, thereby activating inflammation and promoting chronic inflammatory diseases such as fibrosis and tumors (Berg, 2011; Bueno et al., 2019; Li et al., 2019). For instance, chronic high ethanol exposure exceeded the hepatic metabolism threshold, triggering extensive mitophagy dysfunction and mitochondrial DAMP (mtDAMP) cytoplasmic leakage, thus promoting hepatitis and fibrosis (Zhong and Lemasters, 2018; Subramaiyam, 2023). Enhancement of BNIP3-mediated mitophagy in hepatic macrophages suppressed the leakage of mtDNA into the cytoplasm, the cGAS/STING/NLRP3 signaling pathway, and hepatic stellate cells (HSC) activation, ameliorating hepatic fibrosis (Wang Q. et al., 2022). Additionally, MAVS has been shown to connect the innate immune Nod1/RIP2 signaling pathway, inflammation, and mitochondrial homeostasis by regulating mitophagy, playing a crucial role in cardiac remodeling (Lin et al., 2020).

In summary, mitophagy influences the progression of fibrosis by clearing damaged mitochondria and their pro-inflammatory substances, reducing the activation of inflammasomes, pro-inflammatory factors, and signaling molecules, and inhibiting the innate immune responses. Understanding the interaction between mitophagy and inflammation holds promise for identifying potential therapeutic targets for treating fibrotic diseases.

### 3.3 Mitophagy mediated cellular dynamics regulation in fibrotic diseases

Multiple dynamic changes occur at various stages of the cell life cycle, including cell proliferation, differentiation, and death processes. As a key performer in the regulation of cellular dynamics, mitophagy not only involves the straightforward repair and clearance of mitochondria but also intricately orchestrates biological processes to maintain cellular homeostasis.

The most prominent characteristic of fibrotic diseases is the activation of MFB, whose main sources include differentiation processes such as FB-MFB transition (FMT), EMT, endothelial-mesenchymal transition (EndoMT), as well as fibrocytes derived from the bone marrow (Wynn, 2008). MFB have been identified as a major matrix-producing cell in the fibrosis formation process, participating in wound healing or organ fibrotic processes by secreting ECM protein (Zeisberg and Kalluri, 2013). Therefore, the sustained accumulation of MFB at the site of injury is considered a significant marker of fibrosis (Migneault and Hébert, 2021). TGF-β serves as a key cellular factor in the pathogenesis of fibrosis and exerts a pro-fibrotic effect by inducing Smad-dependent and non-Smad-dependent signaling pathways, promoting FB proliferation, MFB activation, and ECM synthesis (Massagué, 2012; Rockey et al., 2015). Increasing evidence indicates that mitophagy plays a crucial role in regulating MFB activation and is involved in the fibrotic disease process (Figure 2B). Defects in PINK1/Parkin-mediated mitophagy activated platelet-derived growth factor receptor (PDGFR), an important mediator in fibrosis, which promoted FB proliferation and MFB activation, exacerbating fibrosis in IPF, a chronic progressive disease characterized by the fibrosis and hardening of lung tissue; besides, it was noted that Parkin is a major factor regulating the FB phenotype in IPF (Kurita et al., 2017; Tsubouchi et al., 2018). While Pirfenidone exerts anti-fibrotic effects in IPF by enhancing Parkin-mediated mitophagy which inhibits PDGFR-PI3K-Akt axis and TGF-β-induced MFB activation (Kurita et al., 2017). The stress-response protein Sestrin2 enhanced mitophagy and inhibited EMT and ECM deposition by suppressing the TGF-β/Smad signaling pathway, thereby ameliorating DN-associated renal fibrosis (Ala, 2023). Moreover, PM2.5 has been found to promote the activation of HSC and the development of hepatic fibrosis by activating PINK1/Parkin-mediated mitophagy (Qiu et al., 2019).

In addition to modulating cell proliferation and differentiation, mitophagy is also involved in the regulation of cell death (Figure 2C). When stimulated by intense stress, mitochondria release pro-apoptotic proteins, activating the programmed cell death process (Kubli and Gustafsson, 2012). Mitophagy inhibits apoptosis and senescence by eliminating damaged mitochondria, resulting in apoptosis resistance and prolonged survival of cells. In the intrinsic cells of tissues, this process manifests as a protective effect against fibrosis. Studies have indicated that the mechanism of IPF primarily involved defective PINK1/Parkin-mediated mitophagy in AEC, leading to increased occurrence of mtDNA damage, apoptosis, and senescence; these abnormalities collectively led to increased susceptibility to pulmonary fibrosis (Kim et al., 2020; Xiao et al., 2022). Reducing mtDNA damage with the mtDNA base excision repair enzyme also enhanced mitophagy, which in turn attenuated AEC apoptosis and ameliorated IPF (Kim et al., 2020). Skeletal muscle cells (SkMC) can activate, proliferate and differentiate into new muscle fibers to promote repair at injury sites. Enhancement of Parkin-mediated mitophagy in SkMC inhibited senescence-related apoptosis, thereby preventing fibrosis and improving skeletal muscle health (Leduc-Gaudet et al., 2019). Renal tubular epithelial cells (RTEC) contain abundant mitochondria and are susceptible to mitochondrial dysregulation. It has been shown the presence of NLRP3-independent inflammasomes in the kidney, which inhibited mitophagy and enhanced apoptosis in RTEC by binding to MAVS after hypoxic injury, thus promoting renal fibrosis progression (Kim et al., 2018). In mice lacking Caspase-9, enhanced mitophagy reduced apoptosis, attenuated inflammatory response, and ameliorated renal fibrosis by inhibiting the cGAS-STING pathway (Doke et al., 2021). However, when inhibition of apoptosis by mitophagy occurs in macrophages, it manifests as a pro-fibrotic effect. Akt1 enhanced mitophagy and induced the acquisition of apoptosis resistance in alveolar macrophages from IPF patients, thereby contributing to pulmonary fibrosis; conversely, impaired mitophagy induced by knockdown of Akt1 gene enhanced macrophage apoptosis and attenuated TGF-β1 activity, which spared mice from pulmonary fibrosis (Larson-Casey et al., 2016). Apart from apoptosis, ferroptosis is an iron-dependent regulated cell death caused by lipid peroxidation. Recent data showed that FUNDC1-mediated mitophagy led to ferroptosis and hepatic fibrosis by degrading glutathione peroxidase 4 (GPX4) in mitochondria, while knockdown of FUNDC1 ameliorated fibrosis (Bi et al., 2023).

### 3.4 Interaction of mitophagy and energy metabolism in fibrotic diseases

Energy metabolism serves as the foundation for regulating physiological and pathological activities in organisms. Research evidence indicates that the interaction of mitophagy and cellular energy metabolism participates in the fibrotic process (Figure 2D).

On the one hand, when cells perceive energy changes, they can maintain energy homeostasis by regulating the level of mitophagy. AMP-activated protein kinase (AMPK) serves as a critical regulator of energy metabolism that senses and modulates intracellular ATP levels and plays a pivotal role in maintaining mitochondrial homeostasis by regulating mitophagy (Herzig and Shaw, 2018). Activation of AMPKα significantly reduced myocardial injury and fibrosis by promoting mitophagy in acute myocardial ischemia models (Hu J. et al., 2022). Whereas inhibition of the AMPKα1-MFN2 pathway resulted in defective Parkin-mediated mitophagy in NAFLD, which aggravated hepatic fibrosis (Cai et al., 2022).

On the other hand, as the primary energy production center within cells, the functional state of mitochondria directly affects metabolic processes. Existing evidence has suggested that mitophagy maintained energy homeostasis and prevented fibrosis by regulating cellular energy metabolism, particularly glycolysis and lipid metabolism.

Summarizing the relevant literature, we found that mitophagy may interfere with the energy supply and critical life processes of fibrotic cells by altering their glycolysis levels and oxygen consumption; in turn, glycolysis also affects mitophagy levels. A previous study observed increased mitophagy in dermal stromal cells of systemic sclerosis (SSc) mice, with a tendency towards aerobic glycolysis in cellular energy metabolism. This adaptation appeared to be a cellular survival mechanism in response to the pro-inflammatory “hostile” microenvironment. Inhibiting mitophagy and/or aerobic glycolysis was shown to prevent fibrosis in SSc patients (Castello-Cros et al., 2011). Although the above research did not explicitly elucidate the interaction between mitophagy and glycolysis, another study found that activation of Parkin-mediated mitophagy could prevent TGF-β1-mediated FB proliferation and MFB activation by attenuating FB glycolysis process, thus inhibiting fibrosis in IPF (Wang W. et al., 2023). The latest study uncovered reduced expression of acetyl-CoA synthetase short-chain family member 3 (ACSS3) in IPF patients and mice, performed metabolic reprogramming by enhancing anaerobic glycolysis and reducing fatty acid oxidation. This reprogramming process inhibited mitophagy, promoted apoptosis of AEC, and alleviated metabolic imbalance caused by recurrent damage to the alveolar epithelium, ultimately halting the progression of IPF (Wang L. et al., 2023).

The ability of mitophagy to modulate lipid metabolism appears increasingly crucial in metabolic diseases and is closely associated with fibrosis. Liver-specific Parkin gene knockout mice fed a high-fat diet (HFD) exhibited increased hepatic steatosis, along with significant alterations in pathways related to lipid metabolism and fibrosis (Edmunds et al., 2020). This indicates that Parkin-mediated mitophagy may participate in fibrosis by preventing steatosis. Similarly, lysocardiolipin acyltransferase 1 (ALCAT1) has been found to link mitophagy defects to steatosis. Defective Parkin-mediated mitophagy exacerbated hepatic fatty acid oxidation and OS, promoting steatosis and hepatic fibrosis in NAFLD (Wang et al., 2015). Additionally, in HFD-induced diabetic cardiomyopathy models, downregulation of mitophagy led to increased cardiac lipid accumulation, exacerbating myocardial fibrosis (Tong et al., 2019). All these findings suggest that mitophagy is involved in lipid metabolism, and thus protects against fibrosis. Furthermore, it is noteworthy that some scholars proposed that changes in cellular lipid metabolism may occur before activation of mitophagy. A temporal metabolomics study revealed that before PINK1/Parkin-mediated mitophagy reached its peak, lipid metabolism events (e.g., accumulation of triglyceride) occurred and lipid homeostasis was altered in mammalian cells (Long M. et al., 2022). This emphasized the complex interaction between lipid metabolism and mitophagy as well as the importance of considering the temporal relationships between different metabolic processes when delving into the mechanisms of fibrosis.

Taken together, these interacting mechanisms constitute a complex regulatory network, and in-depth studies of the balance between mitophagy and biological processes such as OS, inflammation, MFB activation, apoptosis, and energy metabolism will help to shed light on the pathogenesis of fibrotic diseases and provide new insights for the development of more effective therapeutic strategies.

## 4 Taking mitophagy modulators into consideration for fibrosis treatment

In the current medical field, the prevention and treatment of fibrosis remain highly challenging, with a shortage of clinically available drugs. As mentioned earlier, there is an intimate association between mitophagy and fibrotic diseases, and thus mitophagy modulation holds promise as an effective approach for disease treatment (Suliman and Piantadosi, 2016; Lu et al., 2023). Against this backdrop, we categorized discovered mitophagy modulators into three aspects: natural medicine therapy, biotherapy, and physiotherapy (Table 1). We comprehensively reviewed their therapeutic effects and potential mechanisms in organ fibrosis, providing insights and strategies for exploring new approaches and medications in fibrosis treatment.

**TABLE 1:** Therapeutic effects and potential mechanisms of mitophagy modulators in organ fibrosis.

Category	Subcategory	Name	Target organ	Therapeutic effects on disease	Mechanisms	References
Natural medicine therapy	Natural products	Naringin	Lung	Antioxidant, anti-inflammatory and anti-fibrotic effects on idiopathic pulmonary fibrosis (IPF)	Inhibit ER stress and PINK1-mediated mitophagy Attenuate apoptosis	Wei et al. (2023)
		Farrerol	Kidney	Antioxidant, anti-inflammatory and anti-fibrotic effects	Promote PINK1/Parkin-mediated mitophagy Activate Nrf2 signaling	Ma et al. (2021)
		Quercetin	Kidney	Anti-fibrotic effect	Reduce RTEC senescence via SIRT1/PINK1/mitophagy axis	Liu et al. (2020)
		Poricoic acid A	Kidney	Anti-inflammatory and anti-fibrotic effects on diabetic nephropathy (DN)	Promote mitophagy via downregulation of FUNDC1 Attenuate apoptosis	Wu et al. (2023)
		Oleanolic acid	Heart	Anti-fibrotic effect on age-induced myocardial remodeling Improve cardiac function	FUNDC1-mediated mitophagy	Gong et al. (2022)
		Ginsenoside	Liver	Reduce hepatic steatosis Anti-inflammatory and anti-fibrotic effects (under high-fat diet-induced condition)	Promote mitophagy Inhibit NLRP3 inflammasome activation in macrophages Inhibit HSC activation	Wang et al. (2021)
		Fucoxanthin	Heart	Antioxidant and anti-fibrotic effects on diabetic myocardial fibrosis	Promote Bnip3/Nix-mediated mitophagy Activate Nrf2 signaling	Zheng et al. (2022)
		Chitotriose	Intestine	Antimicrobial, antioxidant and anti-fibrotic effects	Activate Nrf2 signaling Inhibit NF-kB inflammatory pathway and mitophagy Inhibit M2 macrophage polarization and MFB activation	Hu et al. (2022b)
	Traditional Chinese Medicines	Polydatin	Heart	Antioxidant and anti-fibrotic effects (under alcohol-induced condition)	Activate the SIRT6-AMPK signaling pathway Promote PINK1/Parkin-mediated mitophagy	Yu et al. (2022)
		Tongluo Yishen Decoction	Kidney	Antioxidant and anti-fibrotic effects on chronic kidney disease (CKD)	Promote PINK1/Parkin-mediated mitophagy	Jia et al. (2021)
		Number 2 Feibi Recipe	Lung	Antioxidant and anti-fibrotic effects	Promote PINK1/Parkin-mediated mitophagy Activate Nrf2 signaling Attenuate apoptosis	Gu et al. (2022)
		Nuanxinkang	Heart	Anti-fibrotic effect on myocardial infarction (MI)	Promote PINK1/Parkin-mediated mitophagy	Guan et al. (2023)
		Yang-xin-xue keli	Heart	Anti-fibrotic effect on chronic heart failure (CHF)	Scavenge ROS Inhibit mitophagy	Long et al. (2022a)
Biotherapy	Biomolecules	Melatonin	Liver, Kidney, Heart	Antioxidant, anti-inflammatory and anti-fibrotic effects	Promote mitophagy and improve mitochondrial homeostasis Inhibit cell damage, reduce effector cell activation, decrease ECM deposition	Hu et al. (2016) Kang et al. (2016) Yeo et al. (2021)
		Thymosin β4	Lung, Heart	Antioxidant, anti-inflammatory and anti-fibrotic effects (under LPS or H_2_O_2_-induced condition)	Ameliorate defective mitophagy Inhibit oxidative stress damage and inflammasome activation Inhibit TGF-β1-induced FB proliferation and MFB activation	Wang et al. (2022a) Tian et al. (2022)
		Triiodothyronine	Brain	Attenuate neuroinflammation and early brain damage	Promote PINK1/Parkin-mediated mitophagy	Chang et al. (2022)
			Lung	Anti-inflammatory and anti-fibrotic effects (under SiO_2_-induced condition)	Inhibit glycolysis and collagen deposition	Yang et al. (2021)
			Endometrium	Anti-fibrotic effect	Ameliorating the defective autophagy Inhibit EMT of endometrial epithelial cells	Zhou et al. (2022)
		Sestrin2	Kidney	Anti-fibrotic effect on DN	Enhance mitophagy Inhibit TGF-β/Smad signaling pathway, EMT and ECM deposition	Ala (2023)
		MANF	Kidney	Anti-fibrotic effect on renal interstitial fibrosis	Ameliorate defective mitophagy Inhibit cGAS-STING activation	Kim et al. (2023)
		Vaspin	Heart	Anti-fibrotic effect on atrial fibrosis	Promote ULK1/FUNDC1-mediated mitophagy Attenuate apoptosis	Zhu et al. (2022)
		Uridine	Skeletal Muscle	Anti-fibrotic effect on skeletal muscle	Enhance mitochondrial fission and ameliorate defective mitophagy	Dubinin et al. (2022)
	Biomodulators	Rapamycin	Fat graft	Antioxidant and anti-fibrotic effects Increase fat graft survival	Enhance autophagy and mitophagy Attenuate apoptosis	Pei et al. (2023)
		UMI-77	Kidney	Anti-inflammatory and anti-fibrotic effects	Activate mitophagy Attenuate apoptosis and inhibit interstitial inflammation Inhibit TGF-β1/Smad-mediated MFB activation	Jin et al. (2022)
		Finerenone	Kidney	Anti-inflammatory and anti-fibrotic effects	Ameliorate defective mitophagy by PI3K/Akt/eNOS signaling axis	Yao et al. (2023)
		Resmetirom	Liver	Anti-fibrotic effect on non-alcoholic steatohepatitis (NASH)	Enhance mitophagy Promote hepatic lipid metabolism and reduce lipotoxicity	Karim and Bansal (2023)
		JT003	Liver	Anti-fibrotic effect	Activate AMPK signaling Enhance mitophagy Promote collagen degradation	Song et al. (2023)
		Metformin	Kidney	Anti-fibrotic effect	Activate AMPK phosphorylation Promote mitophagy Reduce NLRP3 overexpression	Han et al. (2021)
		PPARδ agonist	Heart	Anti-fibrotic effect on CHF Fail to alleviate cardiac systolic dysfunction	Attenuate over-activated mitophagy	Palioura et al. (2023)
		S-Acetyl-Glutathione	Liver	Antioxidant, anti-inflammatory and anti-fibrotic effects (under CCl4-induced condition)	Ameliorate defective mitophagy	Di Paola et al. (2022)
		Paraoxonase-2	Liver	Anti-inflammatory and anti-fibrotic effects on non-alcoholic fatty liver disease (NAFLD)	Activate mitophagy	Shin et al. (2022)
		MitoQ	Lung, Liver	Anti-fibrotic effect	Enhance mitophagy Reduce mtROS production and mtDNA damage Enhance SIRT1 activity and inhibit cellular senescence	Li et al. (2023b) Turkseven et al. (2020) Zhang et al. (2021)
		SS-31	Kidney	Antioxidant and anti-fibrotic effects on acute kidney injury (AKI)-mediated mesenchymal fibrosis	Promote mitophagy Reduce inflammatory factors expression	Szeto et al. (2017)
		MitoTA293	Lung	Pro-fibrotic effect on IPF	Remove ·OH from mitochondria Inhibit apoptosis and mitophagy in AEC Activate inflammatory and senescence pathways	Sakai et al. (2021)
Physiotherapy		Swimming Exercise	Liver	Anti-fibrotic effect on NAFLD	Activate mitophagy and mitochondrial biogenesis by SIRT1/AMPK signaling axis	Zou et al. (2023)
		Aerobic Exercise	Lung	Antioxidant, anti-inflammatory and anti-fibrotic effects (under aerobic exercise-induced hyperventilation condition)	Increase inhaled PM Inhibit OS, pro-inflammatory responses, and mitophagy	So et al. (2021)

### 4.1 Natural medicine therapy

Natural medicinal therapy refers to various pharmaceutical active ingredients obtained from nature, including natural products from plants, animals, and microorganisms, as well as herbs and compound preparations used in traditional Chinese medical practice, to promote the natural healing and homeostatic regulation of the organism.

#### 4.1.1 Natural products

Natural products are chemical substances produced by organisms in nature, including extracts, endogenous components, or metabolites of animals, plants, and microorganisms. Garnering considerable attention as alternative approaches for disease prevention and treatment, natural products provide strong support for the continuous development of modern medicines.

Plant-derived medicinal natural products, which are generally secondary metabolites of plants, consistently provide numerous highly effective and low-toxicity drug candidates for humans. Recent research highlights the substantial pharmacological effects of plant-derived natural products in improving fibrosis, offering vast prospects for the development of innovative fibrosis treatment strategies. Naringin, a natural product extracted from citrus fruits, inhibited ER stress and PINK1-mediated mitophagy in IPF mice, thereby reducing inflammation and OS levels, decreasing the rate of apoptosis, and protecting lung tissue from fibrosis (Wei et al., 2023). Farrerol, a natural component of Rhododendron with antioxidant and anti-inflammatory activities, protected against renal fibrosis through the activation of Nrf2 and PINK1/Parkin-mediated mitophagy (Ma et al., 2021). Quercetin, a flavonoid compound naturally found in many fruits, vegetables, leaves, and grains, has been shown to alleviate renal fibrosis by reducing RTEC senescence via the SIRT1/PINK1/mitophagy axis (Liu et al., 2020). Poricoic acid A, a natural product isolated from the fungus Poria cocos, has hypoglycemic and antifibrotic properties. It promoted mitophagy via downregulation of FUNDC1, inhibited apoptosis and inflammation, which exerted anti-renal fibrosis effects in DN (Wu et al., 2023). Triterpenoid saponins are medicinal plant-derived natural products and the main active ingredients in many herbal medicines, including oleanolic acid, ginsenoside, and so on. Research indicated that oleanolic acid notably mitigated age-induced myocardial remodeling and improved cardiac contractile function, utilizing the FUNDC1-mediated mitophagy mechanism (Gong et al., 2022). Ginsenoside, the bioactive component of ginseng, alleviated LPS-induced NLRP3 inflammasome activation in macrophages by promoting mitophagy and inhibited HSC activation, thus significantly reducing HFD-induced hepatic steatosis, inflammation, and fibrosis (Wang et al., 2021).

Moreover, natural products derived from marine organisms also participate in the fibrotic process. For instance, fucoxanthin, a marine carotenoid derived mainly from brown algae, has strong antioxidant activity and is widely used in food additives and nutritional supplements. It attenuated myocardial fibrosis in diabetic rats by promoting Bnip3/Nix-mediated mitophagy, enhancing Nrf2 signaling, and mitigating OS (Zheng et al., 2022). Also, chitotriose, a polysaccharide extracted from the shells of crustaceans, has antimicrobial and antioxidant effects. It inhibited NF-kB inflammatory pathway and mitophagy by activating Nrf2, thereby inhibiting M2 macrophage polarization and MFB activation, and ultimately protecting against intestinal fibrosis (Hu K. et al., 2022).

Natural products and their biological functions have become a highly focused research direction in the pharmaceutical industry, with the quantity of relevant scientific studies rapidly increasing. In-depth exploration of the mechanisms by which natural products induce mitophagy is crucial for identifying more potential fibrosis treatment targets, advancing the development of natural medicines, and facilitating their clinical applications.

#### 4.1.2 Traditional Chinese medicines (TCM)

The TCM system is widely utilized globally for various disease treatments with its unique theoretical framework and abundant herbal resources. Research indicated that some herbs and compound preparations can participate in fibrosis treatment by modulating mitophagy. For instance, the herb Polydatin enhanced PINK1/Parkin-mediated mitophagy by activating the SIRT6-AMPK signaling pathway, thereby reducing alcohol-induced OS injury and significantly attenuating myocardial fibrosis (Yu et al., 2022). Compound preparations like Tongluo Yishen decoction, which contains herbs such as Rehmannia glutinosa, Astragalus membranaceus, and Cistanche deserticola, has been widely used to treat CKD as it successfully alleviated renal fibrosis by regulating OS and ameliorating defective PINK1/Parkin-mediated mitophagy (Jia et al., 2021). Number 2 Feibi Recipe, containing ingredients like Astragalus membranaceus and Radix et Rhizoma Salviae Miltiorrhizae, activated PINK1/Parkin-mediated mitophagy and Nrf2 antioxidant signaling in AEC, inhibited apoptosis and OS, thus exerting anti-fibrotic effects (Gu et al., 2022). Likewise, Nuanxinkang, which consists of Panax notoginseng, Salvia miltiorrhiza, and Borneol, also effectively ameliorated myocardial infarction (MI)-related fibrosis by promoting PINK1/Parkin-mediated mitophagy (Guan et al., 2023). All the above-mentioned TCMs work by activating mitophagy, whereas Yang-xin-xue keli, which contains Ginseng, Ophiopogon japonicus, and Schisandra chinensis, reversed the trend of myocardial fibrosis in CHF by scavenging ROS and inhibiting mitophagy (Long K. et al., 2022).

In summary, the complexity of active components in TCM causes diversity in their combined effect results. However, current research on TCM primarily focuses on PINK1/Parkin-mediated mitophagy. To explore innovative treatment strategies, it is essential to conduct more in-depth studies to uncover additional pharmacological effects of TCM on mitophagy, which will provide more possibilities for fibrosis treatment.

### 4.2 Biotherapy

Biotherapy is a modality that utilizes biological self-regulatory mechanisms to achieve therapeutic goals by interfering with the function of biomolecules or introducing specific biomodulators.

#### 4.2.1 Biomolecules

Hormones are chemical messenger molecules secreted by the organism and play crucial roles in physiological regulation, and their involvement in mitophagy to regulate fibrosis has become a hot research topic. Melatonin (MEL) is an indoleamine synthesized and secreted by the pineal gland at night, which has various functions such as regulating circadian rhythms, inducing immune responses, and anti-inflammatory and antioxidant properties (Reiter et al., 2014; 2000; Manchester et al., 2015). In recent years, the anti-fibrotic properties of MEL have attracted much attention, and research has summarized that MEL can exert antifibrotic effects by inhibiting cell damage, reducing effector cell activation, and decreasing ECM deposition (Hu et al., 2016). Moreover, MEL is also synthesized in mitochondria as a mitochondria-targeted antioxidant and has been found to promote mitophagy and improve mitochondrial homeostasis (Tan et al., 2016; Wang et al., 2018). Studies have shown that exogenous supplementation of MEL promoted PINK1/Parkin-mediated mitophagy to remove damaged mitochondria, ameliorating CCl4-induced hepatic fibrosis (Kang et al., 2016), CKD-related renal fibrosis (Yeo et al., 2021) and so on. Therefore, the protective effect of MEL on fibrosis may be closely related to mitophagy. Thymosin β4 (Tβ4) is a protein hormone secreted by the thymus gland with antioxidant, anti-inflammatory and antifibrotic properties. *In vivo* and *in vitro* studies showed that upregulation of Tβ4 in mice significantly ameliorated the defective mitophagy induced by LPS or H_2_O_2_ and exerted protective effects against pulmonary fibrosis and myocardial fibrosis by attenuating OS damage, inhibiting inflammasome activation, and suppressing TGF-β1-induced FB proliferation and MFB activation (Wang F. et al., 2022; Tian et al., 2022). Triiodothyronine (T3), a serum thyroid hormone, has been found to promote PINK1/Parkin-mediated mitophagy, attenuating neuroinflammation and early brain damage (Chang et al., 2022). Besides, T3 exerted protective effects in pulmonary fibrosis and endometrial fibrosis respectively by inhibiting glycolysis to reduce SiO_2_-induced pulmonary injury, inflammation, and collagen deposition (Yang et al., 2021); and by ameliorating defective autophagy to inhibit EMT of endometrial epithelial cells (Zhou et al., 2022). Nevertheless, further studies are needed to explore whether T3 can exert an antifibrotic effect by regulating mitophagy.

In addition, other biomolecules similarly serve to regulate fibrosis by mitophagy. The latest study found that overexpression of mesencephalic astrocyte-derived neurotrophic factor (MANF), a secreted protein belonging to growth factors, ameliorated defective mitophagy and reduced cGAS-STING activation, which inhibited renal interstitial fibrosis (Kim et al., 2023). Adipocytokine Vaspin promoted ULK1/FUNDC1-mediated mitophagy which attenuated apoptosis and atrial fibrosis (Zhu et al., 2022). Chronic treatment with the pyrimidine nucleoside uridine enhances mitochondrial fission and ameliorates defective mitophagy, attenuating fibrosis in skeletal muscle (Dubinin et al., 2022).

#### 4.2.2 Biomodulators

##### 4.2.2.1 Autophagy/mitophagy agonists

Autophagy activator Rapamycin enhanced autophagy and mitophagy, and reduced OS and apoptosis, thereby decreasing fibrosis degree and increasing fat graft survival. Research also emphasized the optimal dosage of rapamycin, indicating the need for a moderate activation of autophagy/mitophagy (Pei et al., 2023). A novel mitophagy activator, UMI-77, alleviated apoptosis of RTEC and interstitial inflammation by activating mitophagy, inhibiting TGF-β1/Smad-mediated MFB activation and hindering renal fibrosis (Jin et al., 2022).

##### 4.2.2.2 Hormone receptor agonists/antagonists

Finerenone, a novel mineralocorticoid receptor antagonist, ameliorated defective mitophagy through the PI3K/Akt/eNOS signaling axis, thereby alleviating renal inflammation and fibrosis (Yao et al., 2023). Completed clinical research has shown that Resmetirom, a thyroid hormone receptor-β agonist, promoted hepatic lipid metabolism, reduced lipotoxicity, and ameliorated NASH-associated hepatic fibrosis by enhancing mitophagy, but peer-reviewed results are still needed to confirm the results (Karim and Bansal, 2023). Recent research has indicated that a dual agonist of adiponectin receptors 1/2, JT003, significantly degraded ECM and ameliorated hepatic fibrosis by activating AMPK signaling and enhancing mitophagy (Song et al., 2023). Similarly, the AMPK agonist, metformin, activated mitophagy and reduced NLRP3 overexpression by inducing AMPK phosphorylation, thereby alleviating renal fibrosis (Han et al., 2021). Furthermore, peroxisome proliferator-activated receptor (PPAR) is a member of the nuclear hormone receptor family, in which PPARδ agonist improved CHF-associated fibrosis by attenuating over-activated mitophagy but failed to alleviate cardiac systolic dysfunction (Palioura et al., 2023).

##### 4.2.2.3 Antioxidants

Studies have shown that some antioxidants not only attenuate OS and cellular damage but also intervene in the regulatory network of mitophagy and participate in fibrosis treatment. For example, S-Acetyl-Glutathione administration restored oxidative homeostasis, ameliorated mitophagy defects, and reduced inflammation, thereby protecting the liver from CCl4-induced fibrosis (Di Paola et al., 2022). Paraoxonase-2, a membrane protein with antioxidant activity, similarly inhibited NAFLD-associated inflammation and hepatic fibrosis by activating mitophagy (Shin et al., 2022).

In addition, some antioxidants specifically target mitochondria, such as mitochondrial quinone (MitoQ), which ameliorated pulmonary and hepatic fibrosis by enhancing mitophagy, reducing mtROS production and mtDNA damage (Turkseven et al., 2020; Li Y. et al., 2023). MitoQ also enhanced the activity of NAD-dependent deacetylase sirtuin-1 (SIRT1) in the manner described above, thereby inhibiting cellular senescence and fibrosis in IPF (Zhang et al., 2021). The antioxidant SS-31 inhibited acute kidney injury (AKI)-mediated mesenchymal fibrosis by promoting mitophagy and decreasing the expression of inflammatory factors such as IL-18 and IL-1β, and this protective effect persisted long after the end of treatment (Szeto et al., 2017). However, a recent study found that MitoTA293, which targeted the removal of intra-mitochondrial ·OH, inhibited apoptosis and mitophagy in AEC, and activated inflammatory and senescence pathways, thus exacerbating IPF; whereas TA293, which targeted the removal of intra-cytoplasmic ·OH, exerted anti-fibrotic effects by inhibiting cellular senescence (Sakai et al., 2021).

### 4.3 Physiotherapy

Physiotherapy is the use of physical means, such as specific sports, to improve health and disease symptoms. As a non-pharmacological intervention, it shows potential in modulating mitophagy to ameliorate fibrosis. A study on zebrafish found that swimming exercise activated mitophagy and mitochondrial biogenesis through SIRT1/AMPK signaling axis, thereby ameliorating NAFLD-associated fibrosis (Zou et al., 2023). Another exercise experiment on mice found that inhaled PM triggered OS, pro-inflammatory responses, and enhanced mitophagy, thus exacerbating pulmonary fibrosis. However aerobic exercise-induced hyperventilation, although increasing the amount of inhaled PM, protected against the above adverse effects (So et al., 2021).

## 5 Summary and outlook

When tissues undergo local damage or stress, substantial cellular responses such as OS, inflammation, and cell death occur locally, subsequently, MFB are activated under the influence of cytokines. Activated MFB participate in wound repair by secreting collagen and reconstructing ECM. However, if the stimulation persists or is widespread, it can lead to excessive repair responses, resulting in the pathological manifestation of fibrosis (Rockey et al., 2015; Xu et al., 2020). As the ultimate pathological outcome of numerous chronic diseases, fibrosis has become a crucial link that urgently needs to be addressed.

With the in-depth exploration of the mitophagy mechanism, researchers have discovered that LC3 protein can recognize ubiquitin chains on the OMM through LC3 adaptor proteins or directly identify and bind to mitochondrial membrane protein receptors with LIR motifs. This guides the autophagosome encapsulation and engulfment of damaged mitochondria. In recent years, the relationship between mitophagy and cellular biological processes such as OS, inflammation, and metabolism during fibrosis has become research flashpoints. However, most related mechanistic studies focus on the PINK1/Parkin pathway, with relatively fewer studies targeting other mechanisms. Therefore, further exploration of potential mitophagy regulatory factors (proteins or noncoding RNAs, etc.) is needed to uncover mitophagy mechanisms in diseases, providing a theoretical basis for further research on therapeutic strategies for these diseases.

It is necessary to note that fibrosis is a complex process involving interactions between various cell types, signaling pathways, and molecular mechanisms. The trends and effects of mitophagy in this process may vary depending on the affected site, pathological stage, and triggering factors. In some fibrotic diseases, increased mitophagy may inhibit molecular events like OS, inflammation, and innate immunity, maintain energy balance and homeostasis, thus suppressing fibrosis, which suggests that mitophagy may be a protective measure taken by cells in response to stimuli such as stress/metabolic imbalance. In some diseases, the occurrence of fibrosis is related to mitophagy dysfunction, possibly because the extent of damage exceeds the protective threshold of mitophagy, causing damage to autophagy-related pathways/proteins and promoting fibrosis development. Furthermore, mitophagy can have different effects on fibrosis by inhibiting/activating MFB activation and suppressing apoptosis in different types of cells. Therefore, further in-depth research is needed to better understand the regulation and mechanisms of mitophagy in different fibrotic diseases. In recent years, mitophagy has also garnered widespread attention in cancer research (Panigrahi et al., 2020). Given that some fibrotic diseases are considered precancerous lesions, it raises the question of whether regulating mitophagy could inhibit the transition from fibrosis to cancer.

Currently, there is a shortage of effective drugs for treating fibrotic diseases. Research has found that natural drugs, biologics, exercise methods, and other regulatory approaches mainly improve fibrosis by affecting PINK1/Parkin-mediated mitophagy. However, there are still limitations in exploring the mechanisms of these methods, and their clinical translation remains a significant challenge in the future. There is an urgent need to find novel regulators that specifically target mitophagy without affecting other organelles or cell activities. This precise control of the biological processes of fibrotic cells will greatly enhance the clinical application potential of mitophagy regulators. Emerging technologies such as artificial intelligence can be used for virtual drug screening, further promoting the development of novel drugs derived from natural products, biologics, and other sources (Xie et al., 2022).

## 6 Conclusion

In this review, we explored the mechanisms of mitophagy and its intricate relationship with the key processes involved in fibrosis formation and summarized the application of mitophagy modulators in fibrosis. This work not only enhances the understanding of mitophagy, but also establishes a vital theoretical foundation for the future development of innovative strategies for treating fibrosis. However, further studies are anticipated to delve even deeper into the mechanisms and regulatory approaches of mitophagy.
